# Epidemiology of *Streptococcus pyogenes* upper respiratory tract infections in Poland (2003–2017)

**DOI:** 10.1007/s13353-024-00875-y

**Published:** 2024-05-17

**Authors:** Izabela Sitkiewicz, Anna Borek, Monika Gryko, Aneta Karpińska, Aleksandra Kozińska, Katarzyna Obszańska, Joanna Wilemska-Dziaduszycka, Jarosław Walory, Agata Bańska, Katarzyna Belkiewicz, Małgorzata Foryś, Agnieszka Gołębiewska, Waleria Hryniewicz, Marcin Kadłubowski, Marlena Kiedrowska, Anna Klarowicz, Bożena Matynia, Patrycja Ronkiewicz, Katarzyna Szczypa, Izabela Waśko, Monika Wawszczak, Izabela Wróbel-Pawelczyk, Bartłomiej Zieniuk

**Affiliations:** 1 Department of Biochemistry and Microbiology, Warsaw University of Life Sciences-SGGW, Nowoursynowska 159, 02-776 Warsaw, Poland; 2 National Tuberculosis and Lung Diseases Research Institute, Płocka 26, 01-138 Warsaw, Poland; 3 National Medicines Institute, Chełmska 30/34, 00-725 Warsaw, Poland; 4 Institute of Biochemistry and Biophysics, Polish Academy of Sciences, Pawińskiego 5A, 01-106 Warsaw, Poland; 5 ALAB Laboratory, Mikrobiologia, Ul. Stępińska 22/30, 00-739 Warsaw, Poland; 6 Centre of Quality Control in Microbiology (Polmicro), Rydygiera 8, 01-793 Warsaw, Poland

**Keywords:** *Streptococcus pyogenes*, Upper respiratory tract infection, Typing, Antibiotic resistance

## Abstract

*Streptococcus pyogenes* (group A *Streptococcus*, GAS) is a major human pathogen and causes every year over 600 millions upper respiratory tract onfections worldwide. Untreated or repeated infections may lead to post-infectional sequelae such as rheumatic heart disease, a major cause of GAS-mediated mortality. There is no comprehensive, longitudinal analysis of the M type distribution of upper respiratory tract strains isolated in Poland. Single reports describe rather their antibiotic resistance patterns or focus on the invasive isolates. Our goal was to analyse the clonal structure of the upper respiratory tract GAS isolated over multiple years in Poland. Our analysis revealed a clonal structure similar to the ones observed in high-income countries, with M1, M12, M89, M28, and M77 serotypes constituting over 80% of GAS strains. The M77 serotype is a major carrier of erythromycin resistance and is more often correlated with upper respiratory tract infections than other serotypes.

## Introduction

*Streptococcus pyogenes* (group A *Streptococcus*, GAS) is a major human pathogen and causes over 600 million upper respiratory tract onfections yearly worldwide. Untreated or repeated infections may lead to post-infectional sequelae such as rheumatic heart disease, a major cause of GAS-mediated mortality (Sims Sanyahumbi et al. 2016; Carapetis et al. 2005).

The significant evolutionary success of this pathogen depends on the plethora of encoded virulence factors such as adhesins, proteases, DNAses, superantigens, and other factors that influence the human immune system (Sitkiewicz 2018). The major adhesin and GAS virulence factor is the M protein. The N-terminal region of the M protein is hypervariable, and the sequence of this fragment allows the assignment of GAS into so-called M types that are equivalent to the serotype.

Serotype distribution differs markedly in various countries. In the USA and high-income countries, strains that belong to the top 10 serotypes constitute usually 70–80% of all isolates. The most prevalent M types in high-income countries belong to M12, M1, M28, and M89 types. Remarkably, the distribution of GAS M types in the Pacific region or Africa is much more variable, and 25 most prevalent serotypes contribute to about 60% of isolated serotypes (Steer et al. 2009). In recent years, multiple genomic analyses have shown mechanisms of clonal expansion and selection of GAS strains either by the acquisition of the DNA via the horizontal transfer or by mutations that may affect virulence (Nasser et al. 2014; Zhu et al. 2015; Beres et al. 2016; Fittipaldi et al. 2012; Carroll et al. 2011; Olsen et al. 2010).

There is no comprehensive, longitudinal analysis of the M type distribution of upper respiratory tract strains isolated in Poland. Previous reports focus rather on their antibiotic resistance patterns or on the invasive isolates (Szczypa et al. 2006, 2004; Strus et al. 2017; Golinska et al. 2016). Therefore, our goal was to analyse the clonal structure of the upper respiratory tract GAS isolated over multiple years in Poland.

## Materials and methods

### Strain maintenance and re-identification

Strains were collected over a period of 15 years, from 2003 to 2017. All strains sent to the National Medicines Institute (NMI) were plated onto Columbia agar plates with 5% sheep blood (Becton Dickinson) and tested for the presence of the Lancefield antigen with Streptex Rapid Latex Agglutination Test (Thermo Scientific). Strains were grown in liquid Columbia medium with 5% sheep blood and 20% glycerol and kept frozen at − 80 °C. In cases when DNA isolated from a strain did not yield results in downstream molecular typing such as amplification of *emm* gene fragment, strains were re-identified using MALDI-TOF typer (Brucker).

### Epidemiological data

Information about the number and incidence of infections in Poland, including GAS, is subject to reporting by physicians to the Chief Sanitary Inspectorate — Department for Communicable Disease and Infection Prevention and Control. The data were analysed by the Department of Epidemiology and Surveillance of Infectious Diseases, National Institute of Public Health — National Institute of Hygiene. Results of the analysis are published as yearly reports (http://wwwold.pzh.gov.pl/oldpage/epimeld/index_a.html, accessed on 12 September 2023, in Polish). Reports for years 1999–2007 present the incidence of scarlet fever, erysipelas, and pharyngitis. A report from 2008 presents the incidence of scarlet fever, streptococcal sepsis, and erysipelas. Reports from 2009 onwards present the incidence of scarlet fever, the total number of invasive infections, erysipelas (considered an invasive infection by the reports), toxic shock syndrome, puerperal sepsis, and other specified and unspecified.

Strains sent to the NMI contained encoded epidemiological information that did not allow the patient’s identification. The patient’s data was stripped from all identifiers, except age, sex, and the source of isolation (i.e. upper respiratory tract). Ethics approval was not required. The collected strains were analysed retrospectively.

### DNA isolation

DNA was isolated as described previously (Borek et al. 2011)*.*

### Detection of antibiotic resistance determinants

Genes responsible for erythromycin and tetracycline resistance in streptococci, i.e*. mef, erm*, and *tet*, were detected in PCR reactions using primers and conditions described previously (Obszanska et al. 2016)*.*

### MIC

Minimal inhibitory concentration assays for penicillin, tetracycline, erythromycin, and clindamycin were performed for all strains carrying *mef*, *erm* or *tet* genes. The assays were performed according to EUCAST (http://www.eucast.org/clinical_breakpoints/, accessed on 12 September 2023). For strains exhibiting resistance to erythromycin, additional double disc diffusion test was performed to distinguish between iMLS_B_ and cMLS_B_ phenotypes.

### Emm typing

M types were assigned based on a protocol available from the Streptococcus Lab at the Centers for Disease Control (https://www.cdc.gov/streplab/groupa-strep/emm-typing-protocol.html, accessed on 12.09.2023). Acquired sequences were blasted against the emm nucleotide database (https://www2.cdc.gov/vaccines/biotech/strepblast.asp, accessed on 12.09.2023).

### MLST

MLST profiles were determined as described previously (Enright et al. 2001) and compared with *S. pyogenes* MLST database (https://pubmlst.org/spyogenes/, accessed on 12 September 2023).

### Detection of GAS virulence factors

Twenty GAS virulence factors were detected as described previously (Borek et al. 2011, 2012a). For all strains, primer mixes 1–3 were used for the detection; amplification with mix 4 (containing primers detecting *spe*B, *spy*CEP, *scp*A, *mac*, *sic*) was performed only for M1 strains.

### Detection of MGE integration sites (phage profiling)

The detection of mobile genetic integration sites was performed and analysed as described previously (Borek et al. 2011, 2012b)*.*

### MLVF

Multi-locus variable number repeat fingerprinting was performed as described previously (Obszańska et al. 2011, 2012)*.*

### Statistical analysis

The statistical analysis was performed using Prism (GraphPad).

## Results

### Epidemiological situation in Poland based on the state-wide reports vs. independent strain collection

**Fig. 1:**
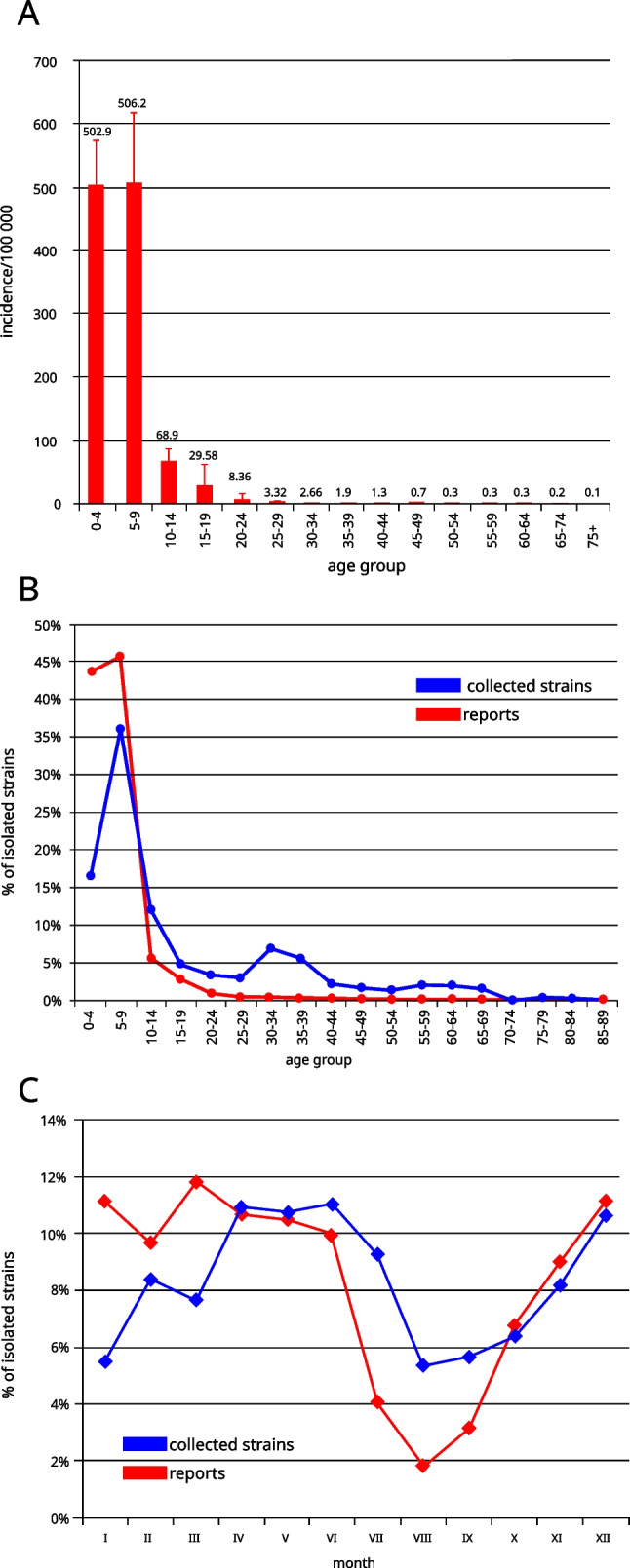
**A** Incidence of scarlet fever in Poland 2003–2017 in age groups based on the data reported by health authorities. **B** Upper respiratory tract infections reported to the authorities and collected isolates (by age groups).** C** Comparison of seasonal variability of infections based on report and strain data

The monitoring of *Streptococcus pyogene*s infections in Poland on the national level is based on the reports submitted by healthcare service providers to the Chief Sanitary Inspectorate. The epidemiological data is then analysed; the incidence ratio calculated and published as bulletins by the National Institute of Public Health — National Institute of Hygiene. The data collection does not require providing the strains for the molecular analysis. Due to voluntary reporting, in many cases, the data suggests misdiagnosis. For example, incidence data suggests overdiagnosis of scarlet fever instead of pharyngitis as the ratios are extremely high in comparison with the data from other countries (Fig. 1A).

Over the years, parallel data has been gathered by the NMI as a part of surveillance programs (http://koroun.edu.pl/koroun/projekt-aleksander/, in Polish). The information about the strains sent to the NMI does not contain the diagnosis, but the source info, i.e. upper respiratory tract or other body sites, information about sex and age of the person who was the source of the strain. Between 2003 and 2017, NMI received 1780 isolates with confirmed upper respiratory tract origin. Strains were isolated from males and females in equal proportions. Strains sent to NMI were predominantly collected from children, and about 65% of strains were collected from children up to 15 years of age. A slight increase was observed for those aged 30–39 what might reflect a group of parents with children aged 0–15 (Fig. 1B). The metadata from the analysis of collected strains reflects multiple epidemiological aspects such as seasonal variability (Fig. 1C), with the highest number of collected strains in April and December and the lowest in August and September.

The strains collected as a part of those surveillance programs were used for molecular analysis to describe molecular dynamics of the *S. pyogenes* population in Poland over 15 years.

### Distribution of M types and association with the respiratory tract infections

**Fig. 2:**
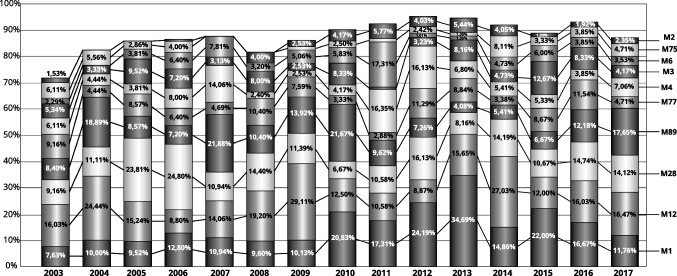
Yearly distribution of 10 the most common M types between 2003 and 2017. As the number of strains sent every year varied, we compared fractions represented by each serotype in a particular year

We assigned M types to 1753 isolates from the upper respiratory tract collected between 2003 and 2017. We detected over 65 different M types; however, 10 M-types constituted almost 88% of all strains (Fig. 2). The largest number of strains belonged to serotypes M1 (287 isolates, 16.4% of collected strains), M12 (283, 16.1%), M28 (235, 13.4%), M89 (187, 10.7%), M77 (129, 7.36%), M4 (120, 6.8%), M3 (113, 6.5%), M6 (82, 4.7%), M75 (58, 3.3%), and M2 (47, 2.7%).

**Fig. 3:**
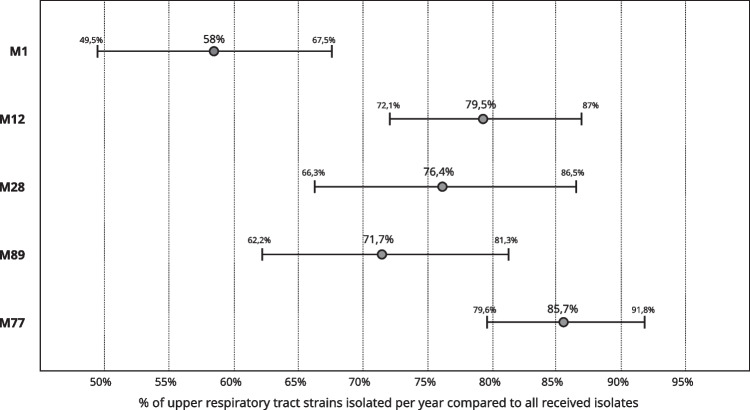
Correlation between the fraction of upper respiratory tract infecting strain compared with for serotypes M1, M12, M28, M89, and M77. Combined data represents years 2003–2017

To test if any particular serotype is responsible for more upper respiratory tract than other types of infections, we performed the correlation analysis for five top serotypes (M1, M12, M28, M89, and M77) (Fig. 3). We compared the year-to-year percentage of strains causing upper respiratory tract vs other types of infections. We did not observe significant differences between M12, M28, and M89 strains isolated from upper respiratory tract vs other available sources (*P* > 0.2). However, a percentage of M1 strains isolated from the upper respiratory tract infections was significantly lower than the percentage of M12 and M28 strains (*P* = 0.0021 and *P* = 0.0149 respectively) that suggests a correlation between M1 serotype and invasive infections. On the contrary, significantly more M77 strains originated from the upper respiratory tract than M89 and M1 (*P* = 0.023 and *P* < 0.0001 respectively).

### Virulence factor distribution

As the virulence of GAS is closely related to the ability of this pathogen to cause the disease, we wanted to test the presence of the virulence factors encoded by upper respiratory tract isolates. However, the presence of certain virulence factors is more related to the invasive disease; we wanted to test the distribution of genes encoding these factors to compare the profiles to profiles from invasive strains in the future. We used a set of multiplex PCR reactions to detected GAS superantigens, DNAses, proteases, and sic gene.

All strains carried at least three virulence genes, with the 80% of strains containing 7–12 virulence factors. We identified only one strain that carried 14 virulence factors. Majority of strains carried *sme*Z, *spe*G, and *sda*B, virtually all strains carried *spe*B, *mac*, and *spy*CEP. *Spe*C was carried by 40% of strains, and other superantigens were encoded by 10–20% of strains, with the smallest fraction carrying *spe*L (70 of 1780 strains). Sic gene was detected in about half of M1 and single M12 strains.

**Fig. 4:**
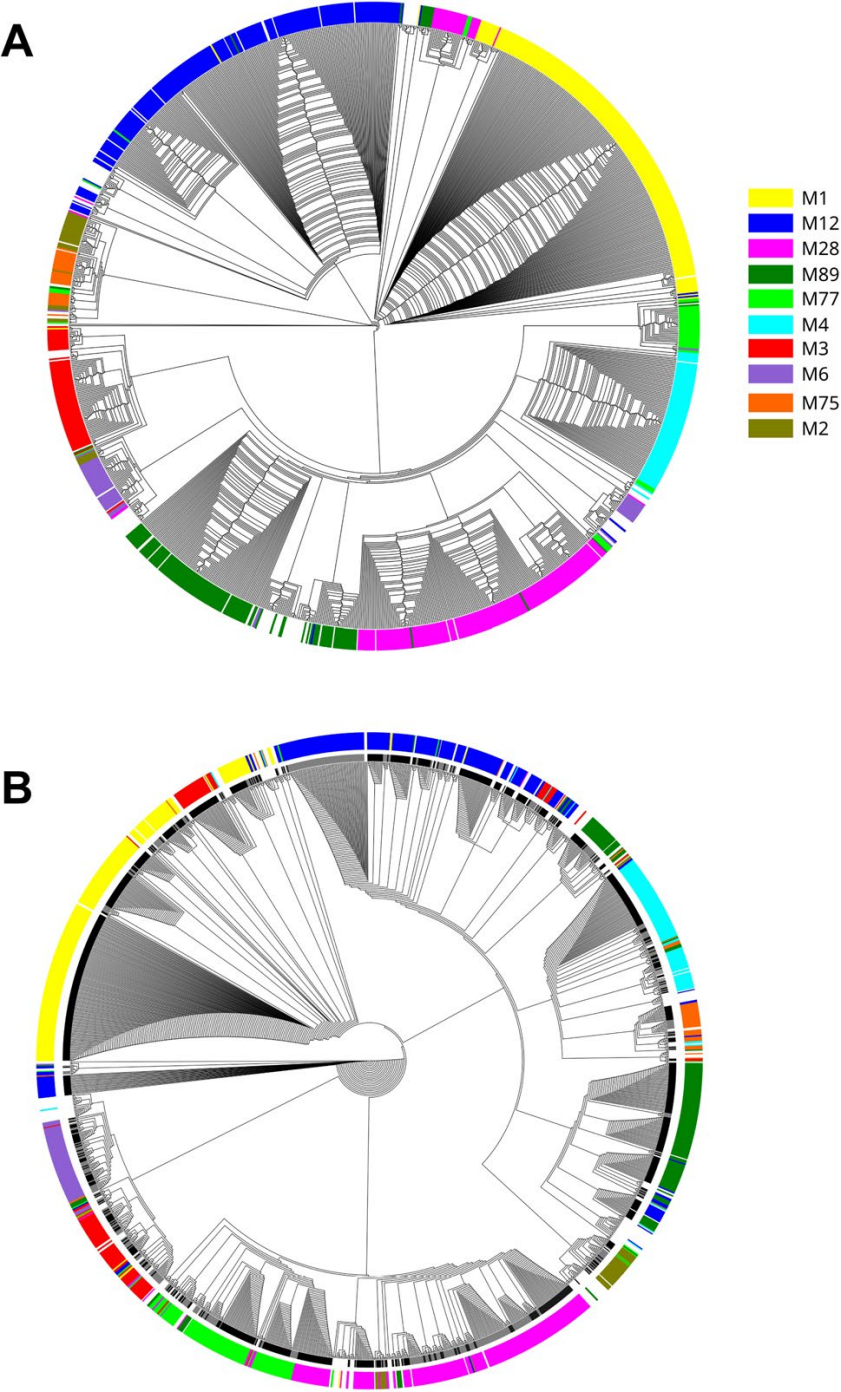
**A** Neighbour-joining tree generated based on the presence of virulence patterns.** B** Neighbour-joining tree based on phage profiling patterns

The analysis of strain similarity based on the presence/absence of the genes encoding virulence factors has shown a close correlation of distinct patterns with the serotype (Fig. 4A). Also typing, based on phage profiling (PP) method, shows correlation between type of PP pattern/type and the serotype (Fig. 4B).

### Evolutionary framework (MLVA based)

**Fig. 5:**
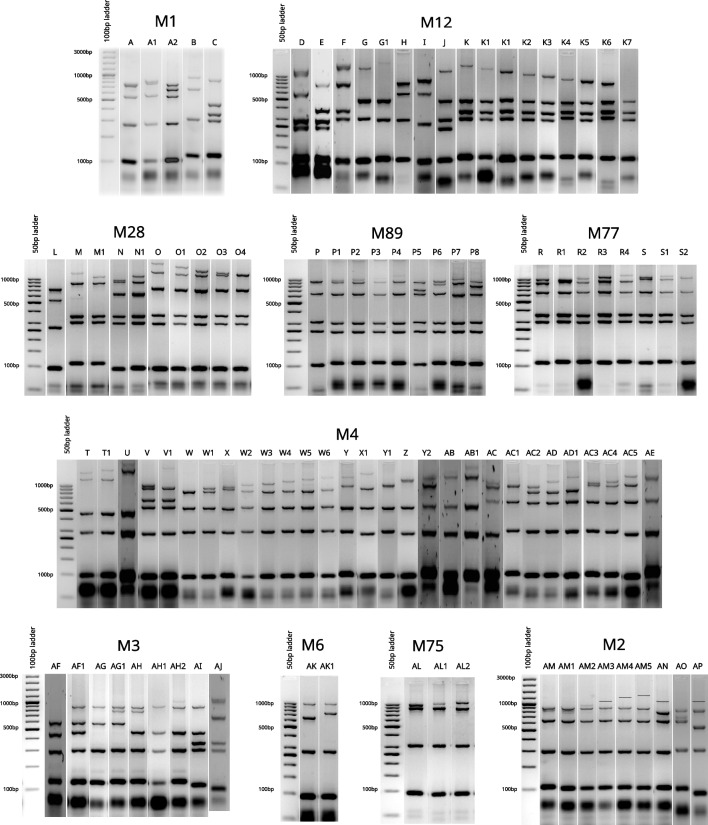
Variability of MLVA patterns detected for 10 the most common serotypes

We performed an analysis of polymorphic loci in GAS genomes (MLVA — multi locus variable tandem repeats analysis) to establish clonal variability of the studied population (Obszańska et al. 2011). Strains of M1 serotype were highly clonal and over 98% of detected MLVA patterns belonged to the single pattern type with subtypes (A-A2). Other serotypes were more diverse, with M12 and M4 strains being the most diverse (Fig. 5).

### Antibiotic resistance

**Fig. 6:**
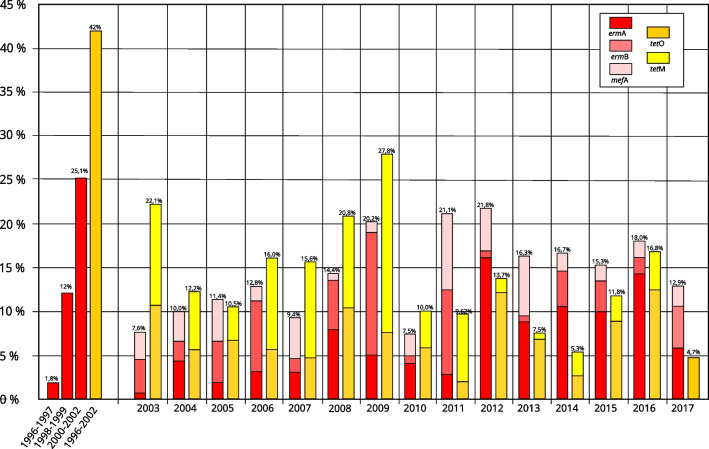
Distribution of erythromycin (red/pink) and tetracycline (orange/yellow) resistance genes by year. Each column shows cumulative fraction that includes *erm*A/*erm*B/*mef*A or *tet*O/*tet*M genes. Data from 1996 to 2002 are after (Szczypa et al. 2004) without resistance gene analysis, shown as comparison to our data; red columns show the phenotypically assessed levels of erythromycin resistance and the yellow one — tetracycline resistance

We screened by PCR all isolates for the presence of genes responsible for the majority of erythromycin (*mef*A, *erm*A, and *erm*B) and tetracycline (*tet*M and *tet*O) resistance in GAS. Three hundred and sixty-seven of 1780 strains carried at least one of the resistance genes (20.6%). Overall, 259 strains carried erythromycin resistance gene (129 *erm*A, 72 *erm*B, 58 *mef*A) and 238 carried tetracycline resistance gene (132 *tet*O, 106 *tet*M), what makes 14.6% and 13.4% respectively. However, the fraction of strains carrying antibiotic resistance genes varied from year to year (erythromycin 7.4 to 21.8%; tetracycline 4.7 to 27.8%) without any noticeable increasing or decreasing trend over the years (Fig. 6). The fraction of *tet*O carrying strains was slightly higher than *tet*M (7.1% vs 6.5%). Among macrolide-resistant strains dominated those carrying *erm*A (6.6% of all strains) in comparison with *erm*B (4.4%) and *mef*A (3.35%).

We noted reported previously specific associations between certain M type and carried antibiotic resistance genes. Average resistance to erythromycin in strains with assigned M type was 14,29% (95% CI 12.72 to 16.02%); however, certain M types exhibited significant (*P* < 0.05) increase or decrease in the fraction of resistant genes in comparison with the population average. For example, over 66%, 65%, and 19.6% of M44, M77, and M28 strains, respectively, were resistant to erythromycin, while only 1% of M3, 2.2% of M89, and around 6% of M1, M6, and M2 were found to be resistant. The fraction of erythromycin-resistant strains carried by other M types was within the 12.7% and 16% range.

*Erm*A gene was detected predominantly among M77 and M28 strains (64% and 9.3% of *erm*A carrying strains respectively). *Erm*B gene was distributed among M28 and M12 strains (46% and 33% of *erm*B carrying strains respectively). Majority of *mef*A carrying strains were of M12, M1, and M4 serotypes (26%, 21%, 19% respectively). On the other hand, 119 of 132 (90%) of *tet*O carrying strains were classified as M77. *tet*M gene was strongly associated with M1 (22.6%) and M28 (18%) strains. The M77 strains that carried *tet*O or *erm*A/*tet*O genes were highly clonal, all belonged to ST63, and they cluster together based on MLVA and phage profiling.

To confirm a resistance phenotype, we performed MIC analysis for penicillin, erythromycin, clindamycin, and tetracycline for every strain with the detected resistance gene. In addition, for every putative erythromycin-resistant strain, we performed double disc diffusion test to detect a phenotype of inducibility for the *erm* gene. MIC assays showed that all analysed strains were sensitive to penicillin. MIC median value for penicillin was 0.015 mg/L (MIC_50_ 0.015 mg/L, MIC_90_ 0.03 mg/L). For tetracycline, the MIC values of the resistant strains were either 32 mg/L or 16 mg/L. For erythromycin, over 85% of strains had MIC values above 16 mg/L, regardless of the gene or inducibility mechanism; however, strains that had lower MIC value exhibited either M or iMLS_B_ phenotype.

## Discussion

### Describing the epidemiological situation in Poland

We present first and such broad analysis of the upper respiratory isolates collected in Poland that. The previous report that included strains isolated from upper respiratory tract were prepared by the NMI laboratories (Szczypa et al. 2004); however, it did not include comprehensive population structure of multiple isolates. In addition, recent reports about GAS infections in Poland predominantly focus on genital tract infections (Golinska et al. 2016; Strus et al. 2009) and a comparison of strains isolated in Poland and Germany from vaginal and upper respiratory tract infections (Strus et al. 2017). Our analysis is therefore a detailed background to study other types of GAS infections.

### Serotype distribution and clonal structure of the GAS population

The serotype distribution and clonal structure of the population are closely related to the distribution in high-income countries. The 10 serotypes, M1, M12, M28, M89, M77, M4, M3, M6, M75, M2, constitute almost 90% of all analysed strains. Interestingly, we observed a high number of M77 strains that belong to ST63, carry a similar set of virulence factors (Fig. 4) and share a close similarity of MLVA patterns (Fig. 5). The most clonal serotype was M1; we observed that almost 99% of strains exhibited the same or highly similar MLVA pattern (A, A1-2). On the other hand, strains of M12 serotype (comparable number of analysed strains) showed eight different MLVA patterns, with additional eight sub-patterns (Fig. 5, K-K7). We observed the highest level of diversity among M4 strains. Such differences in diversity are only partially reflected by ST distribution of strains that belong to these serotypes. For example, almost 90% of M1 and M12 belong to a single sequence type — ST28 and ST36 respectively. In the case of M4 strains, STs are predominantly divided between ST15, ST28, ST77, and ST142. The analysis of MLST database https://pubmlst.org/spyogenes/ shows the most variability for M89 strains that is not reflected in our data. However, genomic analysis of 1200 M89 strains shows a diversity caused by horizontal transfer events that produced clones with increased ability to cause infections leading to epidemic waves (Beres et al. 2016). M1 strains are highly clonal as shown by genomic sequence comparison, and their clonality is driven by spread of a dominant M1T1 clone (Nasser et al. 2014). There is not much genomic data about the diversity of M4 strains, but interestingly, they have an important trait — lack of a capsule encoding operon *has* (Flores et al. 2012).

The clustering of strains using detection of virulence factors shows quite uniform distribution of virulence factors within serotypes, what suggests clonal spread of strains within serotypes (Fig. 4A). On the other hand, the detection of mobile genetic element integration sites shows higher variability and is probably a result of independent insertion/excision of mobile genetic elements into strains within serotype (Fig. 4B).

### Antibiotic resistance

Our analysis revealed variable rates of tetracycline and erythromycin resistance over the years (Fig. 6). Previous analyses of the strains collected in Poland between 1996–2002 and 2002–2004 describe a very high (43%) rate of tetracycline resistance among *S. pyogenes* strains (Szczypa et al. 2004; Skoczyńska et al. 2007). However, Szczypa and co-workers analysed combined upper respiratory tract, invasive, and skin isolates. In our study, we detected lower overall resistance to tetracycline. We did not perform the detection of *tet*K and *tet*L genes that can be responsible for the efflux-mediated resistance to tetracycline in group A *Streptococcus* (Speer et al. 1992). However, the data from MIC assays performed at The National Center for Bacterial Meningitis (Koroun) show a declining rate of tetracycline resistance from 20.9% (2002–2004), 20% (2006–2008) to 12% (2012–2016) (http://koroun.edu.pl/koroun/projekt-aleksander/), which suggests that the efflux mechanism does not play a significant role in the resistance and the resistance is caused by ribosome protection mechanisms mediated by *tet*O and *tet*M.

Previously published reports pointed to the rapid increase in macrolide resistance between 1996 and 2002 from 1.8 to 25% and 8.9% (Szczypa et al. 2004; Skoczyńska et al. 2007). The year-to-year variability observed in our study is consistent with the variable resistance levels observed before. The percentage of the resistant strains was consistent with previous reports of antibiotic resistance in neighbouring countries. Also, the dominant phenotype of the erythromycin resistance detected in the region is MLS_B_ (Gracia et al. 2009).

The previous report by Szczypa and co-workers (Szczypa et al. 2004) pointed to the presence of two widespread clones resistant to antibiotics that belonged to ST367 (M44) and ST63 (M77) and exhibited two types of PFGE patterns. In our study, those M types also constituted a majority of erythromycin-resistant strains and phage profiling and MLVA typing showed that these strains are highly clonal.

The analysis of other European populations shows that the distribution of erythromycin/tetracycline resistance among GAS varies; certain clones are common for many regions. For example, the analysis of Serbian strains not only showed the domination of M75/*mef*A/ST49 and M12/*mef*A/ST36, but also detected third major emm77/ermA/tetO/ST63 clone widespread in Poland (Opavski et al. 2015).

Despite clear therapeutic recommendations for the antibiotic prescription for the upper respiratory tract infections (Hryniewicz et al. 2009; Emeryk et al. 2016), primary care physicians in Poland tend to prescribe and overuse antibiotics for sore throats (Chlabicz et al. 2008). Available data about macrolide consumption/prescription in Poland (Wojkowska-Mach et al. 2018; Chlabicz et al. 2014) suggests the need of proper antibiotic stewardship, and the level of the physicians training and knowledge of upper respiratory tract infections caused by GAS seems to be insufficient (Mazińska and Hryniewicz 2017). Some publications report as high as 38.8% of macrolide prescription rate in cases of severe cough or suspected lower respiratory tract infections (Godycki-Cwirko et al. 2011). Also, the use of macrolides in clinical practice seems to drive the raise if ketolide resistance in Europe (Richter et al. 2008).

### Reporting and nationwide surveillance

The reporting of infections in Poland to the Sanitary Chief Inspectorate does not require strain collection and molecular analysis (EPIMELD database https://wwwold.pzh.gov.pl/oldpage/epimeld/index_p.html#01). Due to poor reporting and classification of invasive vs. non-invasive disease, until that point is not satisfactory. The number of reported upper respiratory tract onfections seem to reflect the epidemiological situation, but the number of invasive diseases reported in Poland markedly differs from those reported for other countries.

Such type of statistical data gathering without strain collection does not reflect the epidemiological situation. Over the years, we observed the improvement of the reporting that better reflects the number of upper respiratory tract infections in comparison with the invasive infections. However, the diagnosis and data collection require education and a unit or reference centre that coordinates epidemiological analyses, as the surveillance should be enhanced by molecular strain analysis. It is clearly visible with increased number of scarlet fever infections worldwide.

## Data Availability

The link to Polish EPIMELD database that collects epidemiological data was added.
